# 852. Bridging the Gap in PrEP Provider Training: An Implementation Science Study

**DOI:** 10.1093/ofid/ofab466.1047

**Published:** 2021-12-04

**Authors:** Aditi Ramakrishnan, Jessica Sales, Micah McCumber, Matthew Psioda, Leah Powell, Anandi N Sheth

**Affiliations:** 1 Emory University School of Medicine, Atlanta, GA; 2 Emory University, Rollins School of Public Health, Atlanta, GA; 3 Department of Biostatistics, Collaborative Studies Coordinating Center, University of North Carolina at Chapel Hill, Chapel Hill, NC; 4 Department of Behavioral, Social, and Health Education Sciences, Rollins School of Public Health, Emory University, Atlanta, GA; 5 Emory University, Atlanta, GA

## Abstract

**Background:**

Training healthcare providers in a variety of clinical settings to deliver pre-exposure prophylaxis (PrEP) is a key component of the Ending the HIV Epidemic (EHE) initiative. Self-efficacy, the individual’s belief in their ability to carry out the steps of PrEP delivery, is a core part of provider training and necessary for successful PrEP implementation. We characterized self-efficacy among providers from family planning (FP) clinics that do not provide PrEP to inform provider training strategies.

**Methods:**

We surveyed providers (any clinical staff who could screen, counsel, or prescribe PrEP) from FP clinics in 18 Southern states (Feb-June 2018, N=325 respondents from 224 clinics not providing PrEP) using contraception- and PrEP-specific self-efficacy questions (overall and grouped into PrEP delivery steps: screening, initiation, and follow-up). We compared self-efficacy scores (5-point Likert scale) by prescriber status, between PrEP delivery steps, and used linear mixed models to analyze provider-, clinic-, and county-level covariates associated with overall PrEP self-efficacy.

**Results:**

Among 325 FP providers, self-efficacy scores were lowest in the PrEP initiation step, higher in follow-up, and highest in screening (p < 0.0001, Table). Mean overall PrEP self-efficacy scores were significantly higher among prescribers compared to non-prescribers (p < 0.0001). However, providers reported lowest self-efficacy regarding insurance navigation for PrEP with no significant difference by prescriber status. The mixed model demonstrated overall PrEP self-efficacy was positively associated with favorable PrEP attitudes among non-prescribers, PrEP knowledge among prescribers, and contraception self-efficacy in both groups, but was not associated with availability of insurance navigation on-site or other covariates (Figure).

Provider Self-Efficacy along the PrEP Delivery Model stratified by prescriber status

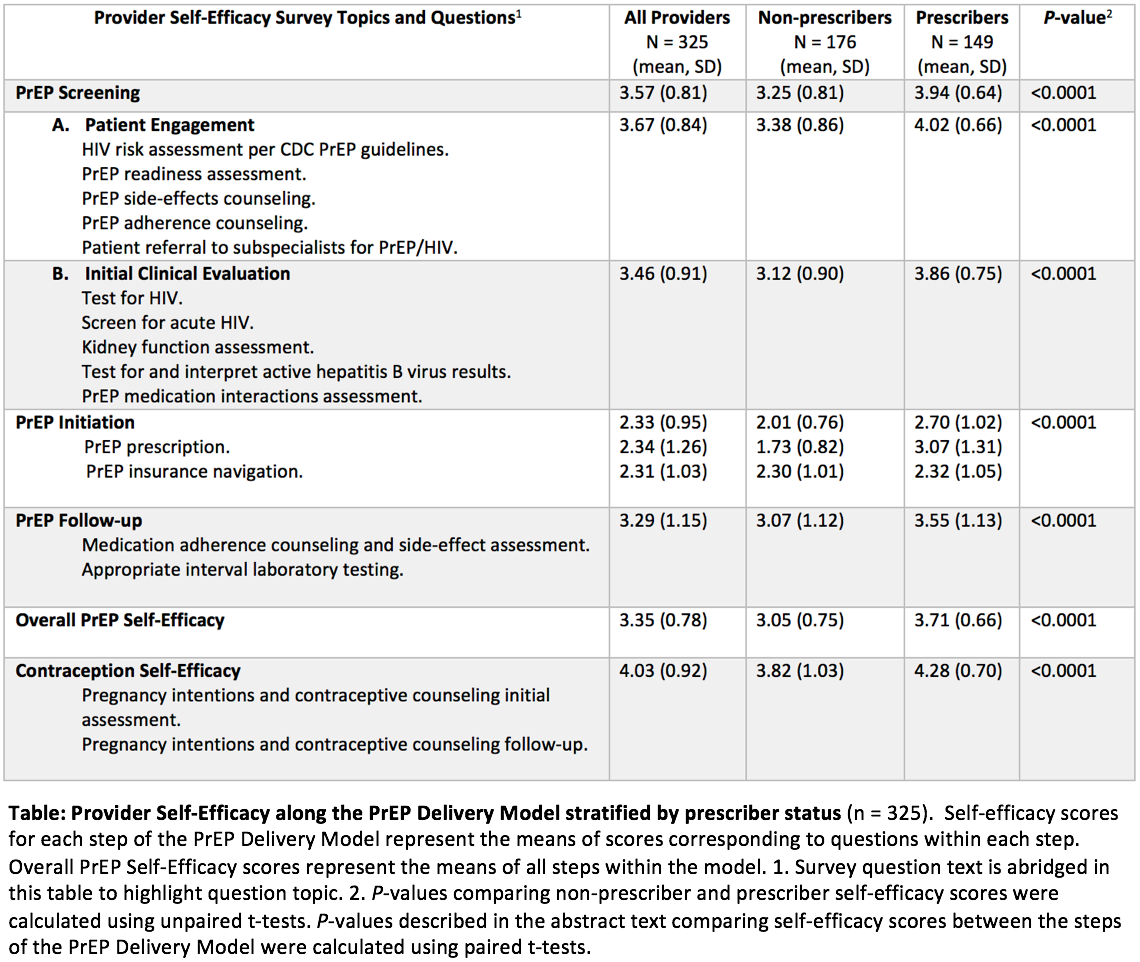

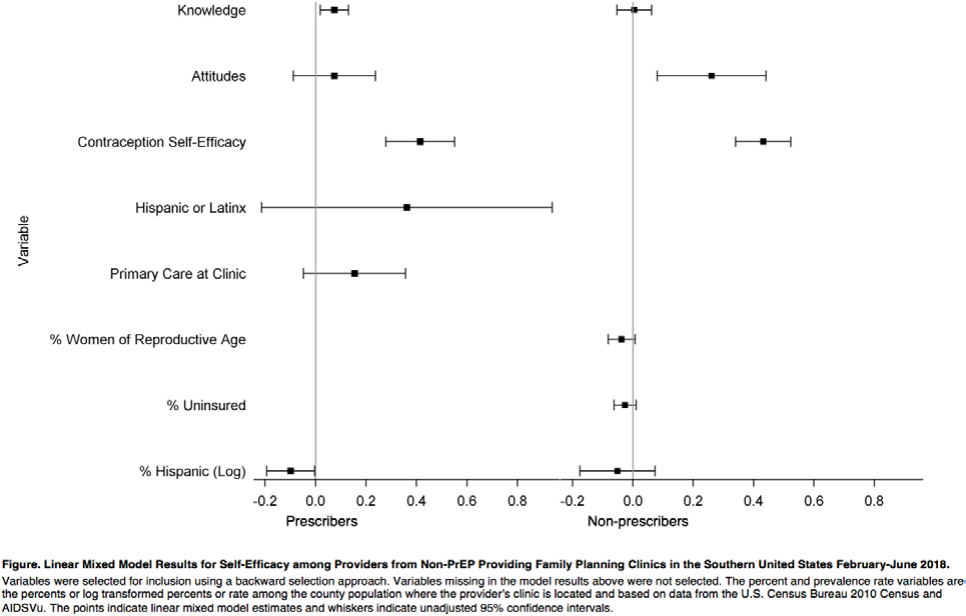

**Conclusion:**

FP providers reported low confidence in their ability to perform the steps that comprise PrEP initiation. Provider training focused on elements of PrEP initiation are critical to improve PrEP implementation and EHE initiatives. Alternatively, programs employing referral or telehealth models to support the PrEP initiation step can successfully bridge this gap.

**Disclosures:**

**All Authors**: No reported disclosures

